# Severe Restrictive Lung Disease in One of the Oldest Documented Males With Coffin-Lowry Syndrome

**DOI:** 10.1177/2324709618820660

**Published:** 2019-01-15

**Authors:** Frederick Venter, Andrew Evans, Claudia Fontes, Carol Stewart

**Affiliations:** 1 Kern Medical Center, Bakersfield, CA, USA; 2 Rio Bravo Family Medicine Residency Program, Bakersfield, CA, USA

**Keywords:** Coffin-Lowry, restrictive lung disease, mental retardation, kyphoscoliosis, Cobb’s angle

## Abstract

Coffin-Lowry syndrome is expressed as different phenotypes in males and females. In males, it is characterized by facial abnormalities, marked developmental disability, and skeletal changes. Approximately 80% of cases are associated with kyphoscoliosis, which can be quite severe, as seen in our patient, causing paraplegia and restrictive lung disease. In this article, we present the third oldest documented male case of Coffin-Lowry syndrome with severe kyphoscoliosis, paraplegia, and restrictive lung disease.

## Introduction

Coffin-Lowry syndrome (CLS) usually presents as facial dysmorphism, psychomotor and growth retardation, digit abnormalities, and progressive skeletal changes as described by Pereira et al.^1^ It was first characterized in 1966 as mental retardation with osteocartilaginous anomalies by Coffin et al,^2^ and later in 1971, Lowry et al again described the condition, focusing on its genetic inheritance pattern.^3^ Temtamy et al further described the syndrome with identification of the histopathologic changes within connective tissue.^4^ The skeletal abnormalities in these patients can be quite severe and exaggerated as seen in our patient leading to paralysis and respiratory complications including restrictive lung disease. Mutations in the *RPS6KA3* gene have been implicated as the underlying genetic cause of CLS and its skeletal abnormalities as a result of impaired osteoblast differentiation.^5^ This condition has been well documented to be inherited in an X-linked fashion.^4^ Newer studies have identified 70% to 80% of cases to be the result of sporadic mutations.^1^

## Case Presentation

**Figure 1.**
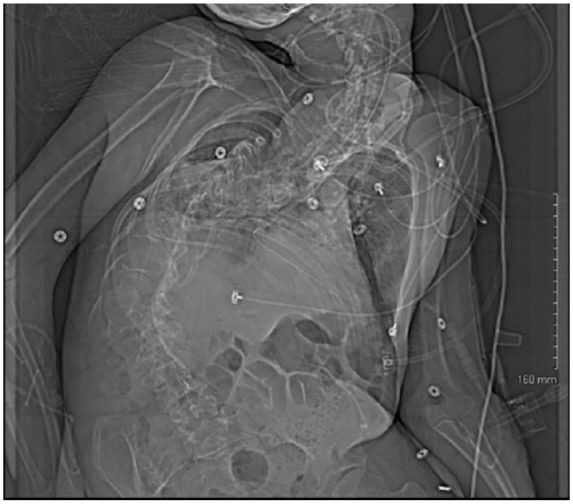
Scout chest X-ray showing severe kyphoscoliosis of patient with Coffin-Lowry syndrome.

**Figure 2.**
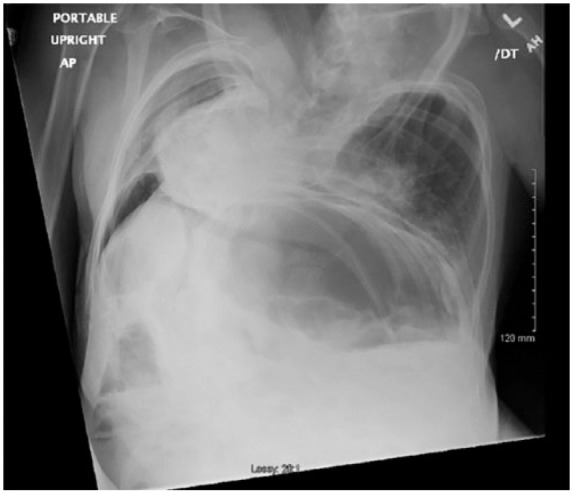
Chest X-ray showing left basilar airspace disease.

**Figures 3 and 4.**
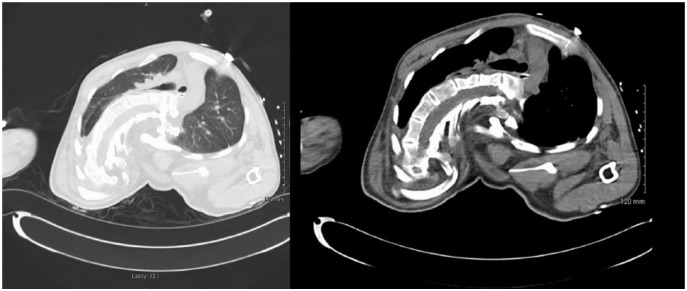
Axial computed tomography scan demonstrating severity of skeletal abnormalities and associated restrictive lung disease.

A 33-year-old male with known CLS presented to the hospital with a 2-day history of cough, hypoxia, and shortness of breath. On admission, the patient’s vitals were significant for pulse rate of 103 beats per minute and oxygen saturation of 88% on room air and an arterial blood gas pH of 7.38 with PCO_2_ 58 and HCO_3_ 34. Physical examination revealed characteristic findings of CLS, including broad nose, large ears, hypertelorism, down-slanted palpebral fissures, oligodontia, pectus excavatum, and severe kyphoscoliosis with decreased breath sounds in the lower lung fields, worse on the right side. The lung examination was limited, secondary to the significant skeletal abnormalities. With concern for aspiration pneumonia, a chest X-ray was ordered, which suggested left basilar airspace disease (Figures 1 and 2). This study was followed by a computed tomography of the chest revealing the extent of skeletal abnormality (Figures 3 and 4).

This patient suffered from undiagnosed chronic respiratory failure caused by restrictive lung disease secondary to congenital kyphoscoliosis. During hospitalization, there was an initial concern for aspiration pneumonia because of leukocytosis and declining respiratory function with a new arterial blood gas pH of 7.23 with PCO_2_ 84 and HCO_3_ 35; however, induced sputum cultures solely grew normal throat flora. Initially, the patient was started on nebulized ipratropium bromide/albuterol but required intubation for declining oxygenation and fatigue. As the patient’s condition improved, he was extubated the following day and managed on BiPAP (bilevel positive airway pressure). Although the patient continued to demonstrate improvement, he required oxygen via nasal cannula after a failed trial on room air. Withholding oxygen for approximately 10 minutes resulted in arterial carbon dioxide and oxygen pressures of 75 mm Hg and 47 mm Hg, respectively, indicating a need for oxygen supplementation. The patient was discharged on 2 liters of oxygen via nasal cannula and BiPAP PRN as recommended by pulmonology. At the 6-month follow-up, patient is reported to be doing well.

## Discussion

This patient’s kyphoscoliosis is so severe, even in terms of CLS, that at the time of this hospitalization he was officially diagnosed with restrictive lung disease causing hypoxemia of 86% on room air. An attempt to measure the Cobb’s angle by our radiologists was proven unsuccessful due to the patient’s inability to stand for an upright chest X-ray due to his paralysis caused by spinal cord compression due to skeletal abnormalities associated with CLS.

Welborn et al found in their cohort study that frequent evaluation of skeletal changes should be carried out by health providers with a lower threshold for surgical intervention to limit spinal cord compression.^6^ His severe skeletal deformities with resultant anatomical flexion, rotation, and contractures created significant difficulty in visualizing where vertebral bodies begin and end, making it impossible to gauge where to draw the necessary Cobb’s angle lines. Moreover, the severity of his scoliosis led to significant difficulties with ventilation.

Proper management of this patient relied on early identification of the patient’s decompensated respiratory status and impending respiratory failure with early intubation. Worsening kyphoscoliosis is observed in most cases of CLS.^5^ Although this patient did not require home oxygen previously, it is suspected that the worsening kyphoscoliosis was the major contributing factor leading to chronic restrictive lung disease and the requirement for home oxygen therapy.

While there is limited data on CLS in patients after the third decade of life,^7^ genetic analysis in a 40-year-old maternal uncle of 2 newly diagnosed males with CLS revealed mutations of the *RPS6KA3* gene.^5^ More than 125 mutations have been identified with the *RPS6KA3* gene, a growth factor-regulated serine-threonine protein kinase, an X-linked disorder in CLS. Some mutations insert or delete genetic material while others change amino acid building blocks, which are responsible for encoding regulatory proteins responsible in signaling pathways within cells controlling growth, division, specialization, and apoptosis of cells causing skeletal and intellectual abnormalities seen in patients with CLS.^8^

This condition affects 1/50 000 to 100 000 people.^9^ The aforementioned patient, however, showed a very mild phenotype of CLS suggesting that this syndrome can present with a wide spectrum of severity.^5^ Although there is no cure for CLS, there should be a focus on symptomatic treatment; we suggest early intervention with physical, speech, and educational therapy along with frequent skeletal surveys assessing the neurological functions of limbs and extremities with a low threshold for surgical intervention to limit spinal cord compression. Our patient is currently 34 years old, making him the third oldest documented male with CLS.^5,10^

## Conclusion

CLS is a well-described coalition of abnormalities that have been attributed to loss-of-function mutations in the *RPS6KA3* gene.^1^ This patient’s degree of kyphoscoliosis at the time of hospitalization resulted in restrictive lung disease. Currently, at 34 years of age, this patient is the third oldest male patient reported with CLS.^5,10^
